# Saltwater intrusion and human health risks for coastal populations under 2050 climate scenarios

**DOI:** 10.1038/s41598-024-66956-4

**Published:** 2024-07-10

**Authors:** William Mueller, Daniel Zamrsky, Gualbert Oude Essink, Lora E. Fleming, Aniruddha Deshpande, Konstantinos C. Makris, Benedict W. Wheeler, John N. Newton, K. M. Venkat Narayan, Abu Mohd Naser, Matthew O. Gribble

**Affiliations:** 1 Institute of Occupational Medicine, Edinburgh, UK; 2 Department of Physical Geography, Utrecht University, Utrecht, The Netherlands; 3 Deltares, Unit Soil and Groundwater Systems, Utrecht, The Netherlands; 4 University of Exeter Medical School, Penryn, UK; 5 Department of Epidemiology, Emory University, Atlanta, GA USA; 6 Cyprus International Institute for Environmental and Public Health, School of Health Sciences, Cyprus University of Technology, Limassol, Cyprus; 7 Division of Epidemiology, Biostatistics, and Environmental Health, University of Memphis, Memphis, TN USA; 8 Department of Medicine, Division of Occupational, Environmental & Climate Medicine (OECM), University of California, San Francisco, CA USA

**Keywords:** Climate change, Salinity, Low- and middle-income countries, Sea level rise, Hydrology, Hypertension, Climate-change impacts

## Abstract

Populations consuming saline drinking water are at greater risk of high blood pressure and potentially other adverse health outcomes. We modelled data and used available datasets to identify countries of higher vulnerability to future saltwater intrusion associated with climate change in 2050 under Representative Concentration Pathways (RCP)4.5 and RCP8.5. We developed three vulnerability criteria to capture geographies with: (1) any coastal areas with projected inland saltwater intrusion of ≥ 1 km inland, (2) > 50% of the population in coastal secondary administrative areas with reliance on groundwater for drinking water, and 3) high national average sodium urinary excretion (i.e., > 3 g/day). We identified 41 nations across all continents (except Antarctica) with ≥ 1 km of inland saltwater intrusion by 2050. Seven low- and middle-income countries of higher vulnerability were all concentrated in South/Southeast Asia. Based on these initial findings, future research should study geological nuances at the local level in higher-risk areas and co-produce with local communities contextually appropriate solutions to secure equitable access to clean drinking water.

## Introduction

According to the most recent report of the Intergovernmental Panel on Climate Change^1^, human emissions of greenhouse gases (GHGs) have led to an average warming of 1.1 °C compared to 1850 to 1900. Temperatures are on track to reach 2.8 °C by the end of the twenty-first century^2^. Environmental and human impacts of climate change will become more severe with further warming, including increased frequency of extreme weather events (e.g., heatwaves, flooding, droughts), ocean acidification, and rising sea levels in certain areas.

One of the significant consequences of the rise in sea level is the contamination of groundwater from saltwater intrusion^3^. This can lead to compromised water quality (e.g., increased salinity) in coastal areas, particularly in low- and middle-income countries (LMICs), where drinking water is more likely to be untreated^4^. ‘Salinity’ refers to the amount of dissolved salts in a body of water, the majority of which include Na, Mg, Ca, and K, among others. Drinking water salinity associated with groundwater aquifer sources is expected to worsen in the future due to greater agricultural demand for groundwater, the increased salinisation of surface waters from anthropogenic sodium sources, and other impacts of global climate change (e.g., more intense droughts)^5^.

Drinking water remains an underestimated source contributing to the total body burden of salts. Salt intake is associated with high blood pressure, a key risk factor for cardiovascular diseases, chronic kidney disease, and dementia^6^. A recent scoping review identified significant associations between drinking water salts and high blood pressure in half the studies reviewed^5^. With more than 600 million people living in coastal areas < 10 m above sea level^7^, this study aimed to identify countries with populations who may be at greater vulnerability to high blood pressure, and the related health risks, from saltwater intrusion under future climate change scenarios.

## Results

**Figure 1:**
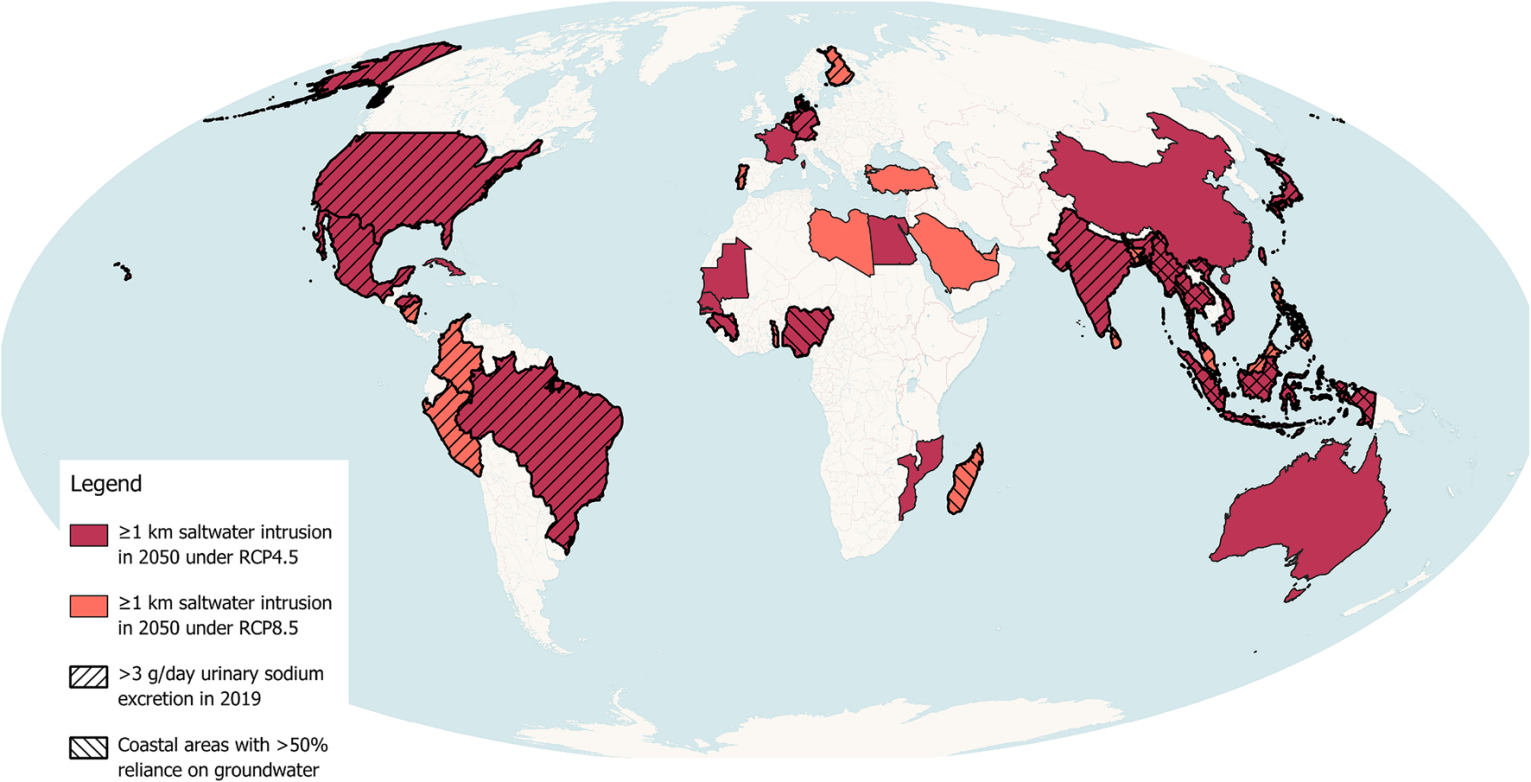
Countries with coastal segments projected to have ≥ 1 km of saltwater intrusion in 2050 according to Representative Concentration Pathways (RCP) 4.5 or 8.5^10^, as well as elevated urinary sodium excretion, and > 50% reliance on groundwater (low- and middle-income countries only). National boundary layer downloaded from opendatasoft < https://public.opendatasoft.com/explore/dataset/world-administrative-boundaries/ > (Mollweide projection).

The global mapping of saltwater intrusion identified 41 countries with at least one coastal segment of ≥ 1 km inland of projected saltwater intrusion in Representative Concentration Pathways (RCP) 8.5 (i.e., ‘very high’ emissions) and 26 countries in RCP4.5 (‘intermediate’ emissions) climate change scenarios (Fig. 1; Table 1). All 26 countries with ≥ 1 km of saltwater intrusion in RCP4.5 were included in the corresponding RCP8.5 list of 41 nations.

**Table 1:** Characteristics of countries with ≥ 1 km of modelled inland saltwater intrusion projected in 2050, in descending total coastal length. Bold countries satisfy all three vulnerability criteria.

Country	Coastal population < 100 km (millions)	Coastal population < 100 km (% of total population)	Saltwater intrusion: total coastal length with ≥ 1 km (km)		Saltwater intrusion: max inland distance (where ≥ 1 km)		Mean coastal access to groundwater for drinking water* (% [SD])^12^	Mean daily urinary sodium excretion (g/day [95% Confidence Interval])^14^
			RCP4.5	RCP8.5	RCP4.5	RCP8.5		
United States of America	124.3	39	625.3	1596.1	6.5	17.2	N/A	3.5 (3.4–3.6)
**Indonesia**	**138.6**	**60**	**671.4**	**1028.7**	**12.1**	**12.3**	**78.2 (16.5)**	**4.1 (3.9–4.4)**
Mexico	17.7	16	465.7	715.6	8.9	15.6	4.1 (3.5)	3.5 (3.3–3.6)
Brazil	69.8	36	210.2	632.6	5.9	6.0	26.8 (10.4)	3.5 (3.3–3.7)
Australia	15.2	70	105.8	519.7	6.2	10.1	N/A	2.9 (2.8–3.1)
Nigeria	18.7	12	382.3	382.3	1.3	7.2	76.7 (12.2)	2.5 (2.4–2.7)
India	166.0	14	269.6	374.9	1.5	5.7	40.5 (21.8)	3.9 (3.8–3.9)
Senegal	5.4	42	220.1	354.0	3.4	8.0	48.8 (31.8)	2.7 (2.6–2.9)
**Vietnam**	**53.3**	**60**	**313.8**	**349.4**	**53.4**	**53.9**	**72.4 (20.4)**	**4.1 (3.9–4.4)**
Cuba	5.8	52	188.8	323.1	20.6	24.5	N/A	2.9 (2.7–3.1)
Mozambique	8.9	38	297.1	297.1	4.5	4.8	44.7 (13.7)	2.8 (3.7–3.0)
Egypt	20.1	24	256.0	256.0	8.5	15.8	0.91 (2.1)	2.5 (2.4–2.7)
Honduras	3.1	41	80.5	232.0	1.0	9.4	48.0 (15.9)	4.0 (3.8–4.3)
**Thailand**	**14.1**	**21**	**50.2**	**229.6**	**1.3**	**2.7**	**82.6 (8.3)**	**4.2 (4.1–4.5)**
Netherlands	12.4	74	51.1	214.0	22.4	36.1	N/A	3.2 (3.1–3.4)
Mauritania	0.7	22	91.5	202.0	3.3	8.7	46.2 (15.4)	2.7 (2.6–2.9)
Peru	14.0	48	N/A	134.6	N/A	6.4	15.3 (7.5)	3.6 (3.4–3.8)
France	16.9	27	125.5	125.5	2.2	3.6	N/A	3.0 (2.8–3.1)
Togo	2.5	37	N/A	116.5	N/A	3.1	79.1 (N/A)	2.7 (2.6–2.9)
Malaysia	16.2	58	N/A	116.5	N/A	3.9	N/A	4.1 (3.9–4.4)
Guinea	1.7	16	113.3	113.3	3.5	5.8	52.6 (20.1)	2.7 (2.5–2.9)
Germany	7.2	9	77.3	100.1	2.0	6.0	N/A	3.4 (3.2–3.6)
Madagascar	9.2	46	N/A	79.1	N/A	2.6	53.5 (5.0)	2.8 (2.7–3.0)
**Bangladesh**	**54.1**	**33**	**N/A**	**70.1**	**N/A**	**1.0**	**84.5 (13.2)**	**3.5 (3.3–3.7)**
Colombia	9.5	21	N/A	64.8	N/A	1.2	43.4 (17.4)	4.7 (4.5–5.0)
Denmark	3.7	67	61.7	61.7	1.8	2.1	N/A	3.2 (3.1–3.4)
**Sri Lanka**	**14.0**	**68**	**N/A**	**61.3**	**N/A**	**1.4**	**60.7 (6.4)**	**4.2 (4.0–4.4)**
Nicaragua	2.2	38	N/A	50.5	N/A	1.3	35.4 (10.1)	4.0 (3.8–4.3)
United Arab Emirates	3.8	81	N/A	47.6	N/A	1.4	N/A	2.8 (2.5–3.1)
Fiji	0.6	72	39.4	39.4	1.5	3.3	N/A	2.9 (2.8–3.1)
Japan	100.3	79	38.7	38.7	1.1	1.7	N/A	4.0 (4.0–4.1)
**Philippines**	**66.0**	**70**	**N/A**	**37.6**	**N/A**	**1.0**	**68.2 (17.8)**	**4.1 (3.9–4.3)**
China	204.6	15	32.8	32.8	2.3	19.4	15.7 (6.5)	7.0 (6.6–7.3)
**Myanmar**	**16.1**	**32**	**30.8**	**30.8**	**1.1**	**2.7**	**84.6 (3.0)**	**4.1 (3.9–4.4)**
Turkey	11.0	15	N/A	28.3	N/A	1.5	N/A	2.1 (2.0–2.2)
Portugal	7.1	67	N/A	27.5	N/A	2.0	N/A	3.5 (3.3–3.7)
Gambia	0.8	49	27.1	27.1	8.2	8.8	25.2 (16.9)	2.7 (2.5–2.9)
Libya	4.4	68	N/A	24.1	N/A	6.1	36.1 (27.7)	2.5 (2.3–2.7)
Finland	2.7	50	N/A	24.1	N/A	3.0	N/A	3.3 (3.1–3.4)
Taiwan	20.5	88	22.9	22.9	2.4	4.5	N/A	3.6 (3.4–3.7)
Saudi Arabia	8.7	33	N/A	21.5	N/A	1.0	N/A	2.6 (2.4–2.8)

*Dataset only includes low- and middle-income countries (LMICs).

The saltwater intrusion map overlaid with data on low- and middle-income country (LMIC) populations utilising groundwater for drinking water and high urinary sodium excretion identified seven countries that may be especially vulnerable in RCP4.5 and/or RCP8.5: Bangladesh, Indonesia, Myanmar, Philippines, Sri Lanka, Thailand, and Vietnam (Table 1). Of the overall 41 nations, the proportion of the population living within 100 km of the coast ranged from 9% (Germany) to 88% (Taiwan), and the total length of coastal segments involving ≥ 1 km of inland saltwater intrusion ranged from 0 km in RCP4.5 (n = 15 countries) to 1596 km (USA) in RCP8.5 (Table 1). The maximum distance of inland saltwater intrusion was modelled up to 53.4 and 53.9 km in Vietnam for RCP4.5 and RCP8.5, respectively (Table 1). Access to treated water in LMICs was lowest in Myanmar (15%) and highest in Egypt (99%), and the average urinary excretion of sodium ranged from 2.1 (Turkey) to 7.0 (China) g/day (Table 1).

## Discussion

To our knowledge, this is the first study to identify countries at risk of health impacts from increased sodium consumption related to saltwater intrusion in future climate change scenarios. We found seven LMICs that were particularly vulnerable when considering modelled saltwater intrusion, reliance on groundwater, and average urinary sodium excretion levels. These nations of higher vulnerability are all concentrated in South/Southeast Asia; however, all continents (except Antarctica) are projected to have coastal areas where there will be at least 1 km of inland saltwater intrusion by 2050.

With high proportions of coastal populations in these countries, increased sodium content in drinking water sources may lead to significant increases in blood pressure at a population level. While individual salt-sensitivity varies, higher dietary sodium could adversely affect the brain, heart, kidneys, and blood vessels^8^. The main health outcome with salinisation of drinking water identified to date is hypertension, with emerging evidence for maternal health also possibly attributed to hypertension (e.g., [pre]eclampsia, infant mortality)^9^.

Our study benefitted from the use of an empirically derived global model on saltwater intrusion^10^, as well as global datasets on drinking water sources^11^ and urinary sodium levels^12^. There are several limitations to consider when interpreting our findings. Our analysis was based on a 2D model, though 3D models would be more reliable in some coastal settings (e.g., in deltaic areas), which, at present, are in development. The 2D representative groundwater models manage to capture inter-regional differences and provide a first-order insight into potential future groundwater salinisation due to sea level rise. However, future studies should strongly consider using 3D groundwater models to capture any local variations in topography and geology, as well as include groundwater extractions and rivers. Groundwater extraction, in particular, should be taken into account, as this may have affected our saltwater intrusion estimates, especially in densely populated areas.

Flooding by seawater (e.g., storm surges, tsunamis) is an additional source of groundwater salinisation either by directly salinising open groundwater wells or by infiltration via saltwater pools formed in the low-lying coastal regions after an overwash event^13^. Other climate factors, such as droughts, can lead to lower groundwater recharge, as well as increased reliance on fresh groundwater resources as a main supply for drinking water during these dry conditions in regions that would normally rely mostly on surface water^14^. We were not able to quantify saltwater intrusion in all geological media (e.g., in karstic regions), thus, our overall modelling estimates are likely an underestimate. In addition to saline drinking water, higher sodium content in soil, exacerbated by climate change, could also contribute to greater future salt intake, which we did not incorporate into our analysis^15^.

The coastal population within 100 km may overestimate the percentage of a population potentially affected by saltwater intrusion, given the projected distances were < 100 km. We did not account for projected population growth, groundwater use, or urinary sodium excretion quantities in 2050. We applied an arbitrarily low threshold of access to treated water (i.e., < 50%) as an indicator to identify countries where drinking water contamination would be most likely to lead to higher salt intake. The dataset we used for reliance on groundwater was limited to LMICs, though some coastal populations in high-income countries may not have full access to treated water, so saltwater intrusion could also be detrimental to human health in any such locations. Moreover, we identified a high income country (USA) with the most coastline of modelled saltwater intrusion in excess of 1 km, so the issue is certainly not confined to LMIC geographies.

Our study was global in scope, with analysis conducted at the country level. In reality, saltwater intrusion will disproportionately impact specific coastal areas and not the entire country equally, which may include population subgroups with more or less vulnerability to higher salt intake. Depending on future emission trajectories, it may not be possible to prevent the contamination of groundwater drinking resources in high-risk areas, but adequate treatment of extracted water and/or provision of clean drinking water would reduce the health risks to resident populations. Adaption to these future conditions might take the form of out-migration or personal-level behavioural choices (e.g., local persons substituting less brackish water sources as available)^16^. Specific interventions have included rainwater harvesting, pond sand filter systems, managed aquifer recharge, and solar-powered desalination plants^9^. Based on our initial results, future research priorities may include the study of geological nuances at the local level in higher-risk areas (also incorporating other potential impacts of climate change), identification of populations who are particularly vulnerable and less adaptive to saltwater intrusion^17,18^, and the co-production of contextually appropriate solutions to secure equitable access to clean drinking water.

## Methods

The IPCC outlines RCP2.6, 4.5 and 8.5 for low, intermediate, and very high emission scenarios^1^. These RCPs correspond to projected warming of 2 °C, 3 °C, and 4 °C, respectively. In this study, we modelled saltwater intrusion by 2050 based only on RCPs 4.5 and 8.5, as it is unlikely that the required immediate and sufficient GHG emission reductions will occur to achieve RCP2.6^1^.

We developed three vulnerability criteria to capture geographies with a high potential for saltwater intrusion, inconsistent access to treated drinking water, and populations with high sodium intake. The salinity intrusion modelling was based on a set of 2D representative regional scale variable-density groundwater flow and transport models (groundwater models) covering coastal regions along the global coastline^10^. These groundwater models only focus on regions with unconsolidated sediment aquifers. The representative 2D groundwater models cover coastal stretches that share similar geological and sedimentary characteristics^19^. Furthermore, to better capture regional differences, we differentiated between deltaic regions with larger hinterland reaches^20^ and smaller coastal stretches that are in general much narrower. The resulting 2D representative profile is a mean representation of regional characteristics, such as topography and geology, as explained below.

Multiple open-source global datasets were used as input into the groundwater models. The surface elevation was based on the GEBCO dataset^21^ and the base of the unconsolidated sediments (and thus the base of the groundwater models) was based on global coastal aquifer thickness estimation^22^. The geological settings within the groundwater models were approximated due to lack of geological data (e.g., bore logs) on a global scale. To overcome this issue, we used a set of conceptual geological scenarios based on global datasets of hydraulic conductivity^23,24^ and geological heterogeneity^25^. The SEAWAT code^26^ was used to simulate the variable-density groundwater flow and transport in the regional scale groundwater models.. By using multiple geological scenarios (24 for each coastal region) we ensured our groundwater saltwater intrusion estimates covered a large span of possible geological conditions in each coastal region. These geological scenarios differ in aquifer-aquitard thicknesses, presence of clay capping layer in the continental shelf domain, and total number of aquifer-aquitard layers present in the model domain. To estimate current groundwater salinity conditions in the coastal regions, we commenced our simulations at the last glacial maximum (approximately 24,000 years ago), assuming a sea level of 130 m below current level. Thus, we could simulate the abrupt past sea level rise and any effects on groundwater salinity. The final groundwater saltwater intrusion was calculated as the mean saltwater intrusion from the whole set of groundwater model estimations for each region. We validated our results by comparing the estimated inland groundwater salinity extent to a set of local salinity measurements collected from peer-reviewed literature (72 studies). This validation indicated our estimates could be off by several kilometres (or even more) in several cases, mostly underestimating the groundwater salinity extent^10^. Further details are provided in Zamrsky et al.^10^.

Secondary administrative areas^27^ with populations who rely on groundwater were defined as those with access to other improved (protected wells and springs, bottled water, rainwater collection, bought water) or unimproved (unprotected wells and springs) drinking water sources using 2017 data (i.e., the sum of ‘w_imp_other’ and ‘w_unimp’^11^). The mean percentage accessing groundwater was calculated across coastal administrative areas in each country, which were identified using a shapefile of the world’s oceans and seas^28^. National estimates of the population within 100 km of coastal areas in 2010 were collected from CIESIN^29^. Urinary sodium excretion was used as a proxy for salt intake^30^. Previous research has used a threshold of average 24-h urinary sodium excretion of > 3 g to constitute high sodium intake^31^.

Our final vulnerability criteria were defined as: (1) any coastal areas with projected inland saltwater intrusion of ≥ 1 km inland based on data we previously modelled^10^; (2) LMIC countries where > 50% of the population in coastal secondary administrative areas rely on groundwater; and (3) high national average sodium urinary excretion (i.e., > 3 g/day) for men and women aged 25 + years (2019 data)^12^. Vulnerable LMICs were identified as nations that satisfied all three vulnerability criteria. Data processing and geospatial analysis were performed using Stata v18^32^ and QGIS v3.30.0^33^.

## Data Availability

The datasets used and/or analysed during the current study available from the corresponding author on reasonable request.
